# 3D-printed gallium-infused scaffolds for osteolysis intervention and bone regeneration

**DOI:** 10.1016/j.mtbio.2025.101524

**Published:** 2025-01-27

**Authors:** Hanrui Xi, Xihao Jiang, Shilang Xiong, Yinuo Zhang, Jingyu Zhou, Min Liu, Zhigang Zhou, Chengyu Zhang, Shiwei Liu, Zhisheng Long, Jianguo Zhou, Guowen Qian, Long Xiong

**Affiliations:** a Department of Orthopedics, Second Affiliated Hospital of Nanchang University, NO. 1 Minde Road, Nanchang, Jiangxi, 330006, China; b Institute of Clinical Medicine, Jiangxi Provincial People's Hospital, The First Affiliated Hospital of Nanchang Medical College, Nanchang, Jiangxi, 330006, China; c School of Energy and Mechanical Engineering, Jiangxi University of Science and Technology, No. 1180 Shuanggang East Avenue, Nanchang, Jiangxi, 330013, China; d Department of Orthopedics, Tenth People's Hospital of Tongji University, Shanghai, 200072, China; e Department of Orthopedics, Huashan Hospital, Fudan University, Shanghai, 200040, China; f Department of Orthopedic, Jiangxi Provincial People's Hospital, The First Affiliated Hospital of Nanchang Medical College, Nanchang, Jiangxi, 330006, China; g Department of Joint Surgery, Ganzhou People's Hospital, No. 16, Mei Guan Road, Zhang Gong District, Ganzhou, Jiangxi, 341000, China; h Institute of Orthopedics of Jiangxi Province, Nanchang, Jiangxi, 330006, China; i Jiangxi Provincial Key Laboratory of Spine and Spinal Cord Disease, Jiangxi, 330006, China; j Institute of Minimally Invasive Orthopedics, Nanchang University, Jiangxi, 330006, China

**Keywords:** Gallium doping, Mesoporous bioglass, Tricalcium phosphate, Osteoporosis, Bone regeneration

## Abstract

Exacerbation of osteolysis in osteoporotic bone defects presents a significant challenge for implant-based treatments. This underscores the urgent need to develop implants that actively mitigate osteolysis while simultaneously promoting bone regeneration. In this study, the osteogenic potential of mesoporous bioactive glass (MBG) and β-tricalcium phosphate (β-TCP) was combined with the anti-bone resorption property of Ga doping. Ga-MBG was synthesized using a self-transformation method and subsequently incorporated into β-TCP at concentrations of 5 wt%, 10 wt% and 15 wt%. Scaffolds were prepared using extrusion-based 3D printing. The cytocompatibility of the composite scaffolds and their regulatory effects on the differentiation of osteoblasts and osteoclasts were systematically examined. In addition, the molecular mechanisms underlying bone regeneration and osteolysis regulation in osteoblasts were explored. Subsequently, cranial defects were repaired in a rat model of osteoporosis to assess the therapeutic efficacy and biological safety of the optimal concentration of the Ga-MBG/TCP composite scaffold. These findings indicated that the 10 wt% Ga-MBG/TCP composite scaffold exhibited excellent biocompatibility, enhanced new bone formation, and effectively mitigated osteolysis. These results provide a foundation for further investigation into the optimal concentration of Ga-MBG implants and highlight their potential application in future therapies for osteoporotic bone defects.

## Graphical abstract

**Figure:**
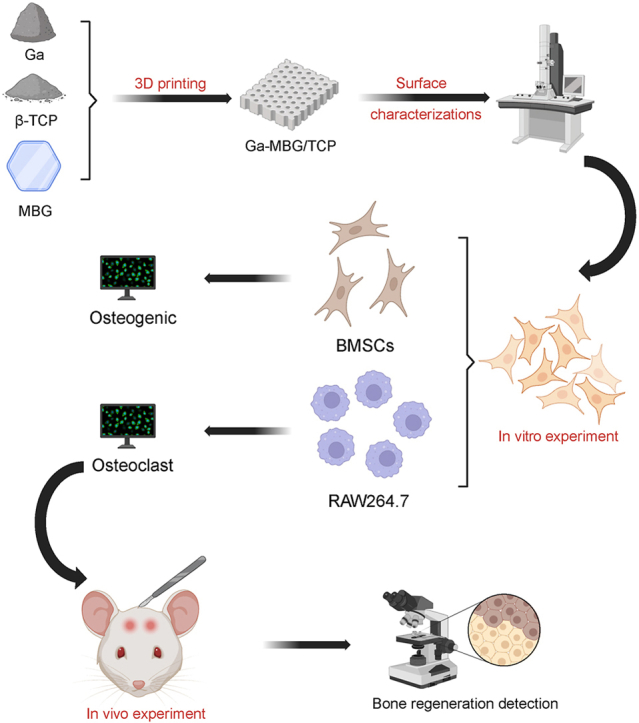

## 1 Introduction

Osteoporosis progressively reduces bone mass and strength, increasing fracture risk [[1], [2], [3]]. Osteoporotic fractures significantly affect patients' quality of life and present treatment challenges, particularly in the repair of bone defects [4,5]. This condition often results in nonunion or delayed healing of fractures, necessitating improved management strategies [6]. To address these challenges, bone repair biomaterials such as β-tricalcium phosphate (β-TCP) and hydroxyapatite have been developed [7,8]. β-TCP is known for its biocompatibility, degradability, and absorption [9]. However, traditional bone repair materials often lack the mechanical properties required for osteoporosis. Excessive strength may inhibit bone remodeling, while insufficient strength increases fracture risk [10]. Unlike traditional materials that mainly enhance bone formation, osteoporotic bone defects require bioactive agents to promote bone growth and reduce bone resorption. Moreover, the degradation rates of conventional materials often fail to align with the healing process of osteoporotic defects. Rapid degradation may hinder repair, whereas slow degradation can obstruct new bone growth and remodeling [11,12].

Due to the limitations of β-tricalcium phosphate (β-TCP), mesoporous bioactive glass (MBG) has gained attention for osteoporotic bone defect repair. MBG offers excellent biocompatibility, promotes tissue integration, and minimizes immune rejection [[13], [14], [15]]. It supports bone regeneration through gradual degradation, promotes osteocyte adhesion and proliferation, and induces mesenchymal differentiation for bone formation [[16], [17], [18]]. Additionally, MBG enhances the release of growth factors such as VEGF, promotes angiogenesis, and accelerates bone healing [19,20]. The mesoporous structure of MBG, characterized by high surface area and large porosity, allows adsorption and loading of metal ions such as silver and strontium. These ions attach to the inner surfaces of MBG pores through physical adsorption and are gradually released into the environment due to their structural characteristics and ion concentration differences [21,22]. In tissue engineering, the controlled release of ions such as zinc and strontium supports bone repair and regeneration. As a scaffold material, MBG continuously releases metal ions, fosters bone cell growth, and enhances tissue repair around implants [23].

Gallium, a metallic element, shares similarities with calcium ions, allowing it to partially substitute for calcium in biological systems and affect bone metabolism [24,25]. Excessive osteoclast activity, which increases bone resorption [26], is inhibited by gallium by targeting specific pathways, including RANKL-stimulated MAPK activation and NF-κB pathways, thereby reducing bone resorption and osteoclast differentiation [27]. It downregulates NFATc1 and inhibits the TRPV5 calcium channel, suggesting its potential as a topical treatment for osteoporotic bone defects [28,29]. However, concerns persist regarding its biocompatibility, as inappropriate concentrations may inhibit osteoblast activity [30,31].

**Scheme 1:**
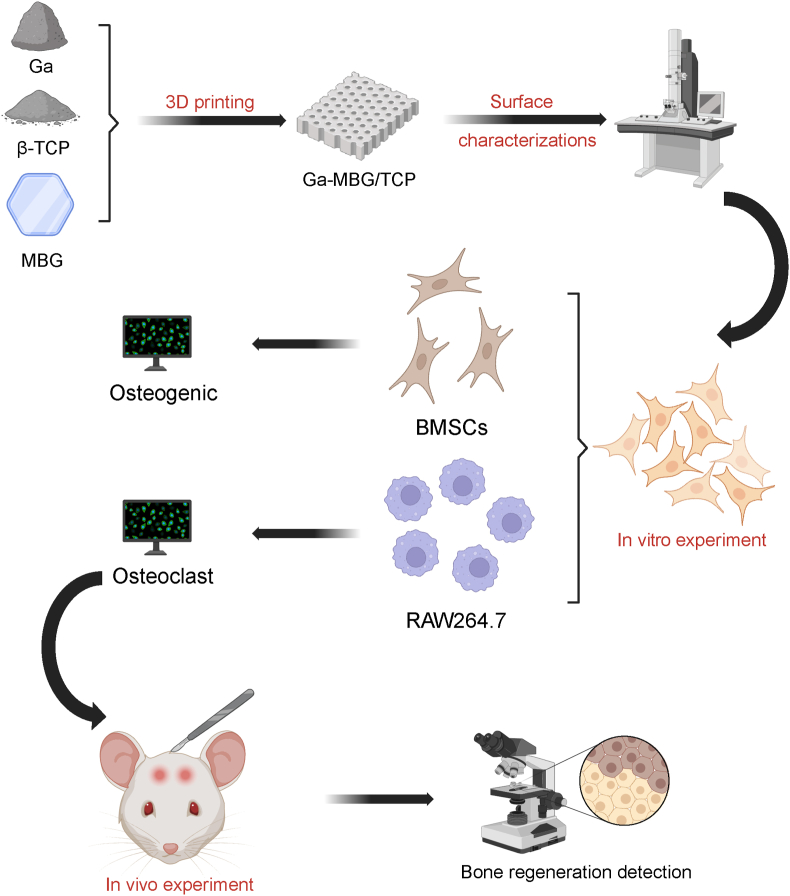
Schematic illustration of the experimental design and procedures (Created with BioRender.com).

Mesoporous bioactive glass (MBG) is particularly effective as a carrier for metal ions, including gallium ions, which exhibit anti-bone resorption effects. The use of MBG as a carrier for Ga enables the sustained release of Ga ions and enhances biocompatibility. β-Tricalcium phosphate (β-TCP) and its derivatives have been successfully applied in clinical practice, providing crucial support for bone tissue repair and stabilizing bone defect sites. Gallium-doped MBG (Ga-MBG) provides additional benefits to this biomaterial system. Combining Ga-MBG with β-TCP further improves the degradation rate and mechanical properties of the composite material, making it suitable for the bone repair process related to osteoporosis. This study investigates the synergistic effect between the anti-bone resorption property of gallium and the bone regeneration capabilities of mesoporous bioactive glass (MBG) and β-tricalcium phosphate (β-TCP). Our objective is to develop an osseointegration strategy that enhances bone regeneration, mitigates osteolysis, and optimizes the microenvironment for bone regeneration and resorption (Scheme 1). Ga-MBG composites were synthesized using a self-conversion technique. To further investigate Ga concentration, scaffolds incorporating 5 wt%, 10 wt%, and 15 wt% Ga-MBG were blended with β-TCP and fabricated using extrusion-based printing techniques. Following the preliminary optimization of their biological activity, we conducted a systematic study on the cytocompatibility of the composite scaffolds and their regulatory effects on osteoblast and osteoclast differentiation. The molecular mechanisms underlying osteogenesis were also examined. Subsequently, the therapeutic efficacy and biosafety of the optimal concentration of the Ga-MBG/TCP composite scaffold were evaluated in a rat model of cranial defects associated with osteoporosis. Based on our findings, the Ga-MBG/TCP composite scaffold, leveraging the bone regeneration capabilities of MBG and TCP, demonstrates potential for treating osteoporotic bone defects.

## 2 Materials and methods

### 2.1 The starting materials

The starting materials included tricalcium phosphate (Ca_3_(PO_4_)_2_, AR, purity≥ 96.0 %), methyl cellulose (450 mPa s) (all from Macklin, China), polyvinyl alcohol (PVA-1799, 98–99 % hydrolyzed), cetyltrimethylammonia bromide (C_19_H_42_BrN, purity≥99 %), aqueous ammonia (AR, 25–28 %), ethyl silicate (≥98.0 %), calcium nitrate tetrahydrate (Ca(NO_3_)_2_·4H_2_O, AR, ≥99.0 %), gallium nitrate hydrate (Ga(NO_3_)_3_·xH_2_O, ≥99.9 %), anhydrous ethanol (CH_3_CH_2_OH) (all from aladdin, China).

### 2.2 Preparation of mesoporous bioglass doped with gallium

**Scheme 2:**
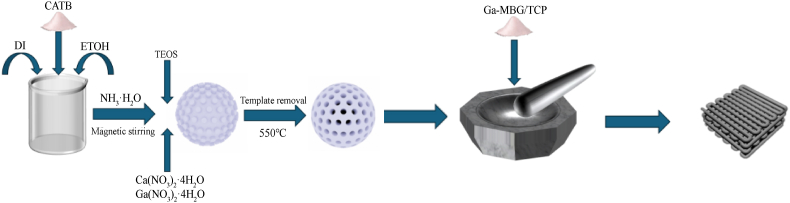
Schematic diagram of the process of powder synthesis and scaffolding (Created with Powerpoint, 3ds Max and Cinema 4D).

The synthesis mechanism of Ga-MBG is illustrated in Scheme 2. MBG materials, with a theoretical SiO_2_:CaO molar ratio of 70:30, were synthesized using a self-conversion method. Tetraethyl orthosilicate (TEOS), calcium nitrate (Ca(NO_3_)_2_·4H_2_O), and gallium nitrate hydrate served as the sources of SiO_2_, CaO, and Ga_2_O_3_, respectively. In this procedure, a beaker was charged with 25 mL of an ethanol solution and 50 mL of deionized water, to which 0.5 g of cetyltrimethylammonium bromide (CTAB, 18 mM) was added under vigorous stirring at 35 °C. Subsequently, 1.5 mL of concentrated ammonia water was introduced into the mixture. Once CTAB was fully dissolved, 2 mL of TEOS was gradually introduced to the solution. After 30 min, 0.871 g of Ca(NO_3_)_2_·4H_2_O and 0.029 g of Ga(NO_3_)_3_·xH_2_O were incorporated into the mixture. The suspension was then subjected to centrifugation at 8000 rpm for 10 min following a 24-h stirring period. Subsequently, the mixture was washed three times with distilled water and absolute ethanol, followed by an additional three washes with deionized water, with centrifugation performed after each washing step. The resulting precipitate was dried at 60 °C for 24 h and then calcined at 550 °C for 6 h, with a heating rate of 5 °C/min. Consequently, MBG was synthesized in a manner analogous to Ga-MBG, with the exception that Ga(NO_3_)_3_·xH_2_O was not included in the process.

### 2.3 Composite scaffold preparation

For the TCP scaffold, 5.5 g of TCP and 0.165 g of methylcellulose were mixed into a mortar, thoroughly ground until completely mixed, and dispersed into 3.67 g of 8 % polyvinyl alcohol solution, and thoroughly stirred with a glass rod to obtain a uniform printing paste. The Ga-MBG was weighed according to 5 wt%, 10 wt%, and 15 wt% of the mass of TCP, respectively, and the remaining steps remained unchanged to obtain 5 wt%, 10 wt%, and 15 wt% Ga-MBG/TCP scaffolds, respectively. A cuboid scaffold model (10 × 10∗2 mm) was constructed by UG modeling software and exported to STL format. Then import the model into UltiMaker Cura 5.3.1 software to slice the model and save it as a gcode file. The software setting parameters are as follows: the filling trace distance is 1 mm, the filling pattern is grid-shaped, the filling trace multiplier is 1, the filling overlap is 100 %, and the filling layer thickness is 0.36 mm. Using an Eazao Bio microfluid bio 3D printer (Chenkun Intelligent Technology, China), nozzle-extruded filaments (diameter 300 μm) were stacked layer by layer along the direction of gravity by controlling the velocity and pressure of the nozzle (0.5 MPa for pressure valve). The material is squeezed out using the force of pressurized air, and the spaces between adjacent filaments form holes for the printed sample. Following the printing of the holder, a 24-h drying period was conducted at room temperature. Subsequently, the holder was placed in a muffle furnace, where the temperature was incrementally increased from 20 °C to 550 °C at a rate of 2 °C per minute over a period of 5 h. The temperature was then further elevated to 1250 °C at the same rate, maintained for an additional 2 h.

### 2.4 Scaffold characterization

#### 2.4.1 Characterization of Ga-MBG nanoparticles

Scanning electron microscopy (SEM; EVO18, ZEISS, Germany) was utilized to investigate the morphological characteristics of MBG and Ga-MBG. The phase composition of MBG and Ga-MBG was analyzed using X-ray diffraction (XRD; D8 Advance, Bruker Co, Germany). Fourier transform infrared (FTIR) spectroscopy (ATR-FTIR; Nicolet 6700, Thermoelectric Corporation) was employed to identify the functional groups present in MBG and Ga-MBG. Additionally, X-ray photoelectron spectroscopy (XPS; Thermo, USA) was conducted to determine the elemental composition of MBG and Ga-MBG.

#### 2.4.2 Physical and chemical properties of Ga-MBG/TCP composite scaffolds

Utilizing a scanning electron microscope (SEM; EVO18, ZEISS, Germany), we conducted an analysis of the microstructure and morphology of composite scaffolds composed of TCP and Ga-MBG/TCP. To ascertain the phase composition of the Ga-MBG/TCP composite scaffold, we employed an X-ray diffractometer. The scaffold was ground into a uniform powder using a mortar to facilitate this analysis. The scaffold dimensions were 10 mm in thickness, 10 mm in width, and 6 mm in height. The materials were evaluated using electronic universal testing machines (Shenzhen Taiyang, China). The tests were conducted at a speed of 1 mm/min for a duration of 40 s to generate load-displacement curves. Subsequently, the compressive strength, compressive modulus, and compressive strain were calculated in accordance with the standards outlined in GB/T 1041-92.

#### 2.4.3 *In vitro* immersion experiment of Ga-MBG/TCP composite scaffold

Inductively coupled plasma atomic emission spectrometry (ICP-AES, Optima 5300DV, PerkinElmer, USA) was used to measure the concentrations of Ca, Ga, Si ions released from four composite scaffolds of TCP, 5 wt% Ga-MBG/TCP, 10 wt% Ga-MBG/TCP, and 15 wt% Ga-MBG/TCP after immersion in phosphate buffer solution (PBS, pH = 7.4) for different times. Specifically, the composite scaffold was immersed in 10 mL of PBS and placed in a vibrating oscillator at 37 °C. and 60 rpm. The weight ratio of the stent to the volume of soaking solution was 16 mL/g. After soaking for a predetermined time, the soaking solution is removed and replaced with a fresh solution. The concentrations of Ca, Ga, Si ions in the soaking solution were determined by ICP-AES.

### 2.5 Determination of biocompatibility

#### 2.5.1 CellCounting Kit-8

A 24 h culture medium was used with a 1 g per 5 mL ratio on the scaffold. In order to evaluate the toxicity of the Ga-MBG/TCP scaffold, bone marrow mesenchymal stem cells (BMSCs) were seeded into 96-well plates with 2 × 10^3^ cells per well. One, two, and three days after harvest, optical density (OD) at 450 nm wavelength (Thermo Scientific, USA) was measured using a miniplate reader (Biosharp, China).

#### 2.5.2 Dead/live staining

Bone marrow-derived mesenchymal stem cells were seeded at a density of 2 × 10^4^ cells per well in 24-well plates and cultured in a medium supplemented with a scaffold. The viability of cells across the three experimental groups was assessed utilizing the Live and Dead Cell Kit (Servicebio, China) by enumerating live and dead cells following 1, 3, and 5 days of culture. Utilize complete medium as the control sample. Fluorescence microscopy was employed to visualize the experimental groups, with calcein AM staining used to identify live cells and propidium iodide (PI) staining used to identify dead cells.

#### 2.5.3 Ratio of hemolysis in blood cells

A single centrifuge tube was prepared containing 100 μL of a 4 % red blood cell suspension derived from healthy Sprague-Dawley (SD) rats. Separate centrifuge tubes were designated for the scaffold extract, phosphate-buffered saline (PBS), and water. The experimental group consisted of centrifuge tubes containing the scaffold extract, while the control group comprised tubes containing PBS and water. The samples were incubated at 37 °C for 1 h to assess the extent of hemolysis. Subsequently, the optical density (OD) of the supernatant was measured at 450 nm using a microplate reader (Thermo Scientific, USA), following careful transfer to a new 96-well plate, to quantitatively evaluate hemolysis. The erythrocyte lysis rate was calculated by the following formula:

$$H\text{emolysis}\;\text{rate}=\frac{\left(\text{OD}1-\text{OD}3\right)}{\left(\text{OD}2-\text{OD}3\right)}\times 100\%$$

In the above example, OD1 denotes a sample test group, OD2 (water) denotes a positive control group, and OD3 (PBS) denotes a negative control group.

### 2.6 The effect of Ga-MBG/TCP composite on osteoblast formation *in vitro*

#### 2.6.1 Differentiation of osteoblasts in cell culture

A 12-well plate seeded with rat bone marrow stromal cells (rBMSCs) at a density of 5 × 10^5^ cells per well was co-cultured with varying concentrations of Ga-MBG/TCP composite. Following a 24-h incubation period, the culture medium was substituted with an osteogenic induction medium supplemented with scaffold extract. This medium comprised Dulbecco's Modified Eagle Medium (DMEM; Servicebio, China), 10 % fetal bovine serum (FBS; Gibco, USA), 10 mM β-glycerophosphate, 10 mM ascorbic acid (Beyotime, China), and 10 nM dexamethasone (all from Beyotime, China). It was replaced every two days for osteogenic induction.

#### 2.6.2 Analyzing and quantifying alkaline phosphatase (ALP)

ALP staining of rBMSCs after 7 days' culture in osteogenic induction medium was followed by microscopy (Leica, Germany) of stained plates. An alkaline phosphatase detection kit (Beyotime, China) was then used to confirm ALP activity, OD510 values were recorded and compared, and alkaline phosphatase content determined using enzyme activity definitions.

#### 2.6.3 A quantitative analysis of Alizarin Red S (ARS) staining

The degree of mineralization in rat bone marrow-derived mesenchymal stem cells (rBMSCs) was evaluated via alizarin red staining 14 days post-osteogenic induction. Following fixation with 4 % paraformaldehyde (Servicebio, China), the cells were stained for 30 min using a 1 % alizarin red solution (Solarbio, China). Images of the stained cells were captured using a light microscope. Subsequently, the optical density (OD) value of the stained cells was measured after treatment with 10 % cetylpyridinium chloride (Aladdin, China).

#### 2.6.4 The immunofluorescence assay of BMP2

The aforementioned protocol was employed to fix the cultured rat bone marrow-derived mesenchymal stem cells (rBMSCs) in 4 % paraformaldehyde at 4 °C following a 7-day culture period. Subsequently, the cells were permeabilized using 0.2 % Triton X-100 (Beyotime, China) for 10 min, followed by a 10-min wash with phosphate-buffered saline (PBS). The cells were then blocked with 5 % bovine serum albumin (BSA) (Servicebio, China) for 60 min. Post-blocking, the cells were incubated overnight at 4 °C with bone morphogenetic protein 2 (BMP2) (ABclonal, China). Finally, the cells were treated with a secondary antibody and incubated for 1 h at room temperature. In the final stage of the procedure, samples underwent staining with rhodamine-phalloidin (Yearsen, China) and DAPI (Abcam, USA) for a duration of 30 min each. Subsequent to observation under a fluorescence microscope (Leica, Germany), the images were processed using ImageJ software (National Institutes of Health, USA).

#### 2.6.5 The expression of osteogenic differentiation-related genes

This study used fluorescence quantitative PCR to analyze mRNA expression of BMP2, RUNX2, and OCN. After osteogenic differentiation of rBMSCs was induced for 7 days, cells were extracted, treated with RNA extract (Servicebio, China) and RNA lysate (Servicebio, China), Nanodrop 2000 was used to detect the concentration and purity of RNA. The reverse transcription reaction system was prepared, and the reverse transcription was completed in a common PCR instrument (Eastwin Life Sciences, China). Finally, the membrane was sealed, and the amplification was completed on a fluorescent quantitative PCR instrument (Biorad, USA), and the results were detected. Table 1 lists the forward and reverse primer sequences from Servicebio. GAPDH levels served as internal controls.

**Table 1:** The primer sequence of all genes used in the real-time PCR.

Gene	Forward	Reverse
Bmp-2	AGGCACCCTTTGTATGTGGACT	GCCTTAGGGATTTTGGAATTCAC
Runx2	TACCCAGGCGTATTTCAGATGAT	TGTAAGTGAAGGTGGCTGGATAGT
OCN	TGACAAAGCCTTCATGTCCAA	CTCCAAGTCCATTGTTGAGGTAG
NFATc1	CACTCCACCCACTTCTGACTTCC	GGCTGCCTTCCGTCTCATAGTG
C-Jun	CCTTCTACGACGATGCCCTC	GGGTCGGTGTAGTGGTGATGT
MITF	GCCCTATGGCTATGCTCACTCTT	TGTTCATACCTGGGCACTCACTC
GAPDH (mouse)	CTGGAGAAACCTGCCAAGTATG	GGTGGAAGAATGGGAGTTGCT
GAPDH (mouse)	CCTCGTCCCGTAGACAAAATG	TGAGGTCAATGAAGGGGTCGT

#### 2.6.6 Migration of osteoblasts

rBMSCs were employed to investigate the impact of gallium ions on cellular migration. The cells were inoculated at a concentration of 1 × 10^5^ cells/mL within the upper chamber of an 8 μm Transwell system (Corning, USA). The corresponding material extracts were introduced into the lower chamber, and the setup was incubated for 24 h. Following incubation, the cells were washed and stained with crystal violet (Solarbio, China) for 1 h, and subsequently examined under a microscope. Furthermore, the cells were also cultured in six-well plates for additional analyses. Once the cells were covered, the serum-free material was replaced to extract the culture medium, and the well plate was uniformly scratched. The cells were cultured for 12 and 24 h, observed under a microscope, and then analyzed with ImageJ.

#### 2.6.7 Transcriptome analysis

One week of culture was carried out with blank control cells and 0 wt% Ga-MBG/TCP composite. RNA-seq samples were processed on the Illumina NovaSeq 6000 after 7 days of cell culture, centrifugation, and TRIzol lysis. Gene expression was quantified by log-transforming RPKM values. Differentially expressed genes were analyzed for KEGG pathway and Gene Ontology (GO) term enrichment using Cluster Analyzer 3.8.1. The STRING database was used to analyze protein-protein interactions (PPI) by building functional protein association networks based on known interactions.

### 2.7 The effect of Ga-MBG/TCP composite on osteoclastogenesis *in vitro*

#### 2.7.1 Differentiation of osteoclasts in cell culture

In this study, RAW264.7 cells isolated from rat bone marrow were used to determine the cytotoxic effect of the material (1 g/5 mL) on RAW264.7 according to previous studies. RAW264.7 cells (1 × 10^5^ cells/ml) in α-MEM (Servicebio, China) with 1 % penicillin/streptomycin (Servicebio, China), 10 % FBS, and 50 ng/mL recombinant rat RANKL (Solarbio, China) were cultured in 24-well plates for 5–7 days, with medium and RANKL refreshed every 2 days. Treatments involved varying concentrations of Ga-MBG/TCP.

#### 2.7.2 Tarrate-resistant acid phosphatase (TRAP) activity evaluation

Following a seven-day co-culture period with varying scaffold concentrations, mature osteoclasts were fixed using 4 % paraformaldehyde for 30 min. A tartrate-resistant acid phosphatase (TRAP) assay kit (Servicebio, China) was used to assess TRAP activity. Light microscopy was used to obtain images of stained cells. In addition, the corresponding cells were extracted, lysed with cell lysate, and then TRAP among the groups was measured using a tartrate-resistant acid phosphatase detection kit (Beyotime, China), OD value was measured at 405 nm absorbance, and TRAP content among the groups was calculated according to the definition of enzyme activity.

#### *2.7.3* The immunofluorescence assays of TRAP and CTSK

After a 7-day culture using the specified method, RAW 264.7 cells were fixed with 4 % paraformaldehyde at 4 °C, permeabilized with 0.2 % Triton X-100 in PBS for 10 min, and blocked with 5 % BSA for 60 min. Thereafter, the cells were incubated overnight at 4 °C with the primary antibody TRAP/CTSK (ABclonal, China). For staining, the cells were treated with rhodamine-phalloidin and DAPI for 30 min post-PBS washing. Fluorescence microscopy was employed for cellular observation, and ImageJ software was utilized for image analysis.

#### 2.7.4 Gene expression associated with osteoclastogenesis

The mRNA levels of osteoclast markers, including C-Jun, MITF, and NFATC1, were measured using fluorescent quantitative PCR. Osteoclastic differentiation of RAW 264.7 cells was induced over a period of seven days, after which the cells were harvested and the aforementioned procedure was replicated. The forward and reverse primer sequences, synthesized by Servicebio, are detailed in Table 1. Gene transcription levels were normalized to GAPDH, which served as the internal control gene.

### 2.8 The therapeutic effect of Ga-MBG/TCP composite stent *in vivo*

#### 2.8.1 Fabrication of animal models

In order to explore the therapeutic effect of Ga-MBG/TCP composite on the skull defect of osteoporotic SD rats, we established the corresponding pathological model of SD rats, and evaluated it by bone mineral density measurement and subsequent radiation and pathology. The study's design and methodology received approval from the Experimental Animal Welfare and Ethics Committee of Jiangxi Provincial People's Hospital (No. NYLLSC2024092902). We used eighteen 8-week-old female Sprague-Dawley rats from GemPharmatech, China, each weighing 200–250 g. In this study, three independent groups (n = 6) were set up, which were 0 wt%, 10 wt% Ga-MBG/TCP and a blank control group. At-60d, rats were anesthetized with an intraperitoneal injection of pentobarbital sodium (1 %, 80 mg/kg), and ovariectomy was performed through a dorsal incision. Bone mineral density was measured five days before the main procedure. Before implantation, the 3D-printed Ga-MBG/TCP composite is shaped into a 2 mm thick, 5 mm diameter circular stent for easy insertion into the bone defect. It is then calcined for shaping, washed with alcohol and saline, and sterilized with UV light. On the day of the procedure, rats were again anesthetized with the same method, and their skulls were exposed via a cranial incision. After the position was determined, the bone fragment was chiseled with a drill bit with an outer diameter of 5.0 mm, and the Ga-MBG/TCP composite material of corresponding size was placed, and the bone fragment was sutured. After normal feeding, rats were intraperitoneally injected with excessive anesthesia (4 % sodium pentobarbital) on 30d and 60d, the skull and various organs were removed, fixed with 4 % paraformaldehyde, and then safety measurement, microscopic ct and histopathological examination were performed.

#### 2.8.2 Determination of biosafety *in vivo*

Drawing upon prior research, we assessed the *in vivo* biosafety of Ga-MBG/TCP composite scaffolds through histopathological analysis of major organs. To examine tissue architecture, the hearts, lungs, spleens, livers, and kidneys of rats in each experimental group (n = 3) were subjected to hematoxylin and eosin (H&E) staining.

#### 2.8.3 Micro-CT experiment

In this study, high-resolution micro-ct (Bruker, Germany) was used to measure the skull at the time of sampling in each group of rats (n = 3), scanned with an isometric resolution of 20 μm and x-ray energy settings of 80 kV and 80 μA. After recording three-dimensional images of the skull, longitudinal and transverse sections were reconstructed and analyzed of the affected regions (ROIs) using the manufacturer's processing software. As part of the reconstructed sections, the following morphometric variables were measured: bone mineral density (BMD), bone volume/total volume (BV/TV), trabecular thickness (TB.TH), and trabecular separation (TB.SP).

#### 2.8.4 Analyses of histological samples

For the decalcification of rat skulls, the specimens underwent a 40-day decalcification process using an EDTA solution (Servicebio, China). Sections were then prepared by slicing around the central axis of the samples and subsequently embedding them in paraffin. These sections were stained using H&E, Masson's trichrome, and TRAP staining techniques. Quantitative analyses were conducted using ImageJ software to assess bone regeneration, calculate the collagen fiber area, evaluate collagen deposition, and determine osteoclast numbers.

#### 2.8.5 Immunofluorescence staining

For immunofluorescence staining, paraffin skull sections were dewaxed and cooled after antigen repair. Tissue was circled with hydrogen peroxide pens, incubated in the dark for 25 min, washed with PBS, and sealed with 5 % BSA for 30 min. Sections were incubated overnight at 4 °C with the primary antibody, followed by a 50-min room temperature incubation with the secondary antibody in the dark. Wash three times, apply TSA (Servicebio, China) dropwise, incubate for 10 min, wash again, perform microwave treatment, add the second primary antibody dropwise, incubate overnight at 4 °C, wash, apply HRP-labeled secondary antibody, incubate for 50 min, add TSA dye, counterstain with DAPI, use autofluorescence quencher B solution (Servicebio, China), incubate for 5 min, rinse, and seal with anti-fluorescence quenching tablet. This study used rabbit anti-BMP-2 and anti-NFATc1 antibodies, both diluted 1:5000, along with HRP-conjugated goat anti-rabbit IgG antibodies (all from Servicebio, China). Immunofluorescence images of BMP-2 and NFATc1 were then taken with a fluorescence microscope (Nikon, Japan).

#### 2.8.6 Immunohistochemical staining

Immunohistochemical analysis was conducted on each skull specimen. Paraffin sections underwent dewaxing, followed by natural cooling post-antigen retrieval. The sections were treated with 3 % hydrogen peroxide to inhibit endogenous peroxidase, washed thrice, and covered with 3 % BSA for 30 min at room temperature. After removing the blocking solution, the primary antibody was applied and incubated overnight at 4 °C. Following three washes, the HRP-labeled secondary antibody was added and incubated for 50 min at room temperature. After washing, a DAB solution was gradually applied to the circle, and the development time was observed under a microscope. A positive result appeared brown-yellow. The sections were then rinsed with tap water to stop the color development, counterstained with hematoxylin, and the plate was dehydrated. The primary antibodies used were rabbit anti-BMP-2 and anti-NFATc1 (both at 1:100 dilution, Servicebio, China), with HRP-labeled goat anti-rabbit IgG as the secondary antibody (1:200 dilution, Servicebio, China). Images were observed and recorded using a Nikon microscope (Japan).

### 2.9 Statistical analysis

Data analysis was performed with SPSS 20.0, using three biological replicates per experiment. Results are shown as mean ± SD, with n = 3. Statistical analysis involved one-way ANOVA and LSD tests, with two-sided significance levels at ∗P < 0.05, ∗∗P < 0.01, ∗∗∗P < 0.001, ∗∗∗∗P < 0.0001, and “ns” for non-significance. P-values less than 0.05 were considered statistically significant.

## 3 Results and discussion

### 3.1 Physicochemical properties of Ga-MBG nanoparticles

The morphology of the MBG and the distribution of Ga were analyzed using the self-conversion method, as shown in Scheme 2. The MBG and Ga-MBG morphologies are shown in Fig. 1 (a) (b), respectively. Scanning electron microscopy (SEM) images show that the prepared MBG and Ga-MBG materials exhibit a spherical shape with rough surface features. The particle size ranged between 0.6 and 0.9 μm, with no significant changes in shape or radius observed after doping gallium. Additionally, some microspheres were crosslinked with each other, forming multispheres with interconnected cavity structures. This morphology suggests that MBG, as a biologically active material with a special pore structure, provides a supporting structure for cell attachment and growth, thereby promoting cell proliferation [32].

**Fig. 1:**
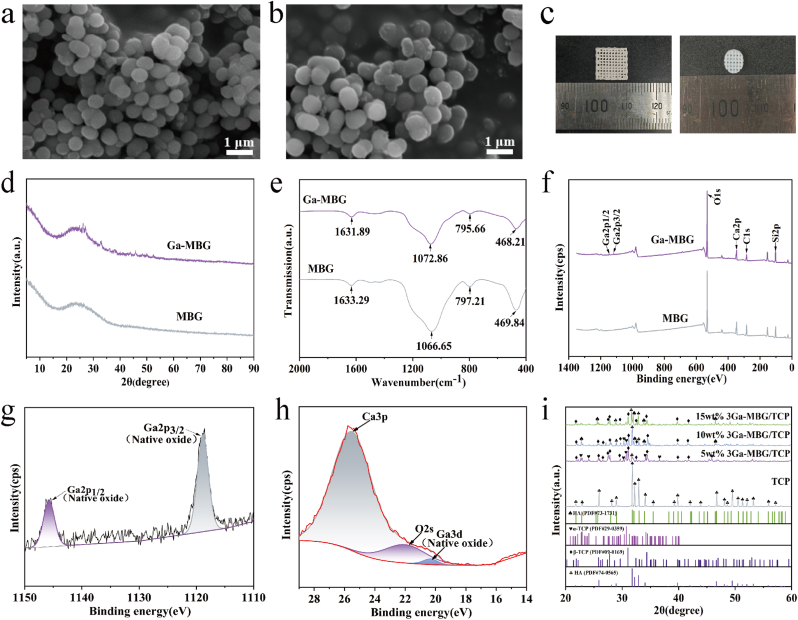
Synthesis and characterization of Ga-MBG/TCP. SEM images of (a) MBG and (b) Ga-MBG. (c) The digital images of the composite scaffolds. (d) XRD patterns of MBG and Ga-MBG powders. (e) FTIR spectra of MBG and Ga-MBG. (f) Full-scan XPS spectra of MBG and Ga-MBG. High-resolution spectra of Ga2p (g) and Ga3d (h) for Ga-MBG. (i) XRD patterns of TCP, 5 wt% Ga-MBG/TCP, 10 wt% Ga-MBG/TCP and 15 wt% Ga-MBG/TCP scaffolds.

X-ray diffraction (XRD) analysis of MBG and Ga-MBG (Fig. 1d) revealed an amorphous structure, indicated by the characteristic broad peak within the 15–35° range [33]. Fig. 1 (e) shows the infrared spectra of MBG and Ga-MBG. The water-related peak at 1632cm-1 and Si–O–Si asymmetric stretching vibrations near 1069cm-1, symmetric stretching vibrations near 796cm-1, and bending vibrations near 468cm-1 were observed. In the Ga-MBG structure, the metal cation affects the Si–O–Si bond angle, causing a band shift in the asymmetric stretching vibration band at approximately 1069cm-1. The presence of smaller metal cations leads to smaller Si–O–Si bond angles. The ionic radius of Ga^3+^ is approximately 76spm, whereas that of Ca^2+^ is approximately 100pm [34]. Due to the smaller ionic radius of gallium ions compared to calcium ions, the addition of Ga^3+^ leads to a reduction in the Si–O–Si bond angle, thereby increasing the asymmetric stretching of Si–O–Si. This increase is evident in the Fourier transform infrared (FTIR) spectrum, where the asymmetric stretching vibration frequency of the Si–O bond is shifted to higher wavenumbers, as shown in figure. X-ray photoelectron spectroscopy (XPS) was used to analyze the composition and chemical states of the elements in the MBG and Ga-MBG nanoparticles. The comprehensive XPS spectra revealed peaks attributable to the constituent elements, facilitating the determination of the relative composition and impurity levels within a binding energy range of 0–1300eV. The C1s peak (hydrocarbon and C–H C–H–C–C) located at 284.6eV was employed to calibrate the measured binding energy (BE) values. Fig. 1 (f) shows two Ga2p peaks and one Ga3d peak, in addition to the expected O1s, Ca2p, C1s, Si2p peaks. The Ga2p peak is shown in Fig. 1 (g), exhibits a high performance-to-noise ratio of its peak was caused by the low Ga content in the Ga-doped MBG. In Ga doped MBG nanoparticles, the Ga2p peaks are observed at 1118.89ev (Ga2p _ 3/2) and 1145.76eV (Ga2p _ 1/2). These peaks represent spin-orbit splitting peaks of the 2p orbital, as reported in previous studies [35]. The Ga3d peak, shown in Fig. 1 (h), appears at approximately 21eV. Binding energies of related peaks include Ca3p at around 25eV and O2s at around 23eV, with the Ga3d and O2s regions clearly overlapping. Due to the higher kinetic energy of Ga3d electrons, the sampling depth is deeper, making it a better reference value for chemical analysis. The Ga3d spectral peaks of elemental gallium are asymmetric, with overlapping spin-orbit components. In contrast, the Ga3d spectral peaks of gallium compounds are symmetric. This observation confirms that gallium exists in compound form rather than as elemental gallium, indicating the successful doping of Ga ions into the MBG matrix.

### 3.2 Physicochemical properties of the Ga-MBG/TCP composite scaffold

The morphology of the Ga-MBG/TCP composite scaffold was examined (Fig. 2a). The figure shows the pore morphology of 3D printed Ga-MBG/TCP bioceramic scaffolds sintered at 1250 °C. The scaffolds had square holes and complete 3D interconnections, created through the stacking and predetermined arrangement of extruded filaments. The pore size of the stents ranged from 350 to 550 μm, while the filament thickness was about 300–350 μm. It is well established that macropores larger than 100 μm are necessary for bone ingrowth, and pores exceeding 300 μm are particularly beneficial for neovascularization [36]. The Ga-MBG/TCP composite scaffold featured interconnected pores of suitable size to support the passage of bone tissue and blood vessels. Fig. 2 (a) shows the microstructures of the scaffold filaments. The tricalcium phosphate particles showed a loose structure with abundant micropores between them [37]. MBG also possesses a distinctive pore structure. Examination of the electron microscopy images revealed that as the content of gallium-loaded mesoporous bioactive glass increased from 5 wt% to 10 wt% to 15 wt%, the microporous structure became more pronounced.

**Fig. 2:**
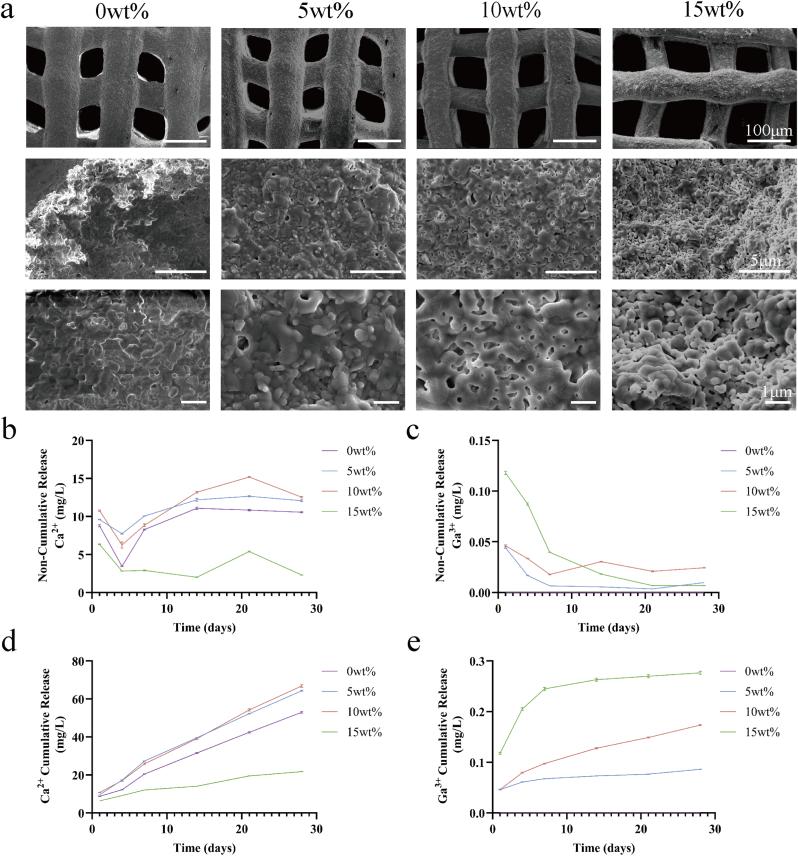
Synthesis and characterization of Ga-MBG/TCP. (a) SEM images of TCP, 5 wt% Ga-MBG/TCP, 10 wt% Ga-MBG/TCP and 15 wt% Ga-MBG/TCP scaffolds. (b) Non-cumulative release curves of Ca^2+^. (c) Non-cumulative release curves of Ga^3+^. (d) Cumulative release curves of Ca^2+^. (e) Cumulative release curves of Ga^3+^.

The phase composition of the composite scaffold is shown in Fig. 1 (i). After TCP was calcined in a muffle furnace at 1250 °C, since the PVA molecular structure contains a large number of hydroxyl groups, and β-TCP provides a source of calcium and phosphorus, they participate in the reaction at high temperatures, providing the conditions necessary for the formation of hydroxyapatite. As can also be seen from the XRD pattern, the β-TCP phase undergoes a transformation and turns into hydroxyapatite. In the 5 wt% and 10 wt% Ga-MBG/TCP samples, both β-tricalcium phosphate (β-TCP) and α-tricalcium phosphate (α-TCP) phases coexisted along with HA. However, in the 15 wt% Ga-MBG/TCP sample, α-TCP was completely absent. Studies have shown that MBG enhances the transition from β-TCP to α-TCP, as bioactive glass contributes to phase stability at temperatures up to 1300 °C. β-TCP remains stable from ambient temperature up to 1125 °C, whereas α-TCP is stable between 1125 °C and 1470 °C. The incorporation of Ga-MBG improved the stability of β-TCP and increased the transition temperature from β-TCP to α-TCP [38]. In the 5 wt% and 10 wt% Ga-MBG/TCP samples, the relatively low doping levels allowed for the coexistence of α-TCP and β-TCP. However, as the doping ratio increased in the 15 wt% sample, the upper temperature limit for α-TCP was surpassed, leading to its absence and the predominance of β-TCP and HA.

The mechanical properties of the scaffolds were tested using a universal testing machine. The compressive stress increased with strain until the yield strength was reached, after which it decreased. As illustrated in Fig. s1, the compressive strengths of scaffolds with 0 wt%, 5 wt%, 10 wt%, and 15 wt% Ga-MBG were about 7.26 MPa, 12.32 MPa, 18.24 MPa, and 26.14 MPa, respectively. Similarly, the compressive moduli were approximately 10.51 GPa, 13.47 GPa, 18.56 GPa, and 24.80 GPa, respectively. These results demonstrate that increasing the doping amount of Ga-MBG enhances the mechanical properties of the scaffolds. When bioactive glass is used as a binding agent, the mechanical properties of β-TCP are improved [38]. Cancellous bone has a compressive strength of 1–12 MPa, while cortical bone is stronger, with a compressive strength of 100–150 MPa [39]. Notably, scaffolds produced via extrusion-based 3D printing possess compressive strengths that exceed those of cancellous bone.

### 3.3 Ion release from the scaffold

Fig. 2 (b) shows the stepwise release of Ca ions from the two scaffold sets. The Ca-ion release profiles of 0 wt%, 5 wt%, and 10 wt% Ga-MBG/TCP scaffolds showed a similar trend. The released amount decreased over the first four days, increased gradually after the fourth day, and stabilized on the fourth day. However, the 15 wt% Ga-MBG/TCP composite scaffold exhibited a slower release of Ca ions, likely due to the excessive Ga concentration, resulting in a lower cumulative release compared to other three scaffolds. The stepwise release of Ga ions from the four scaffolds is shown in Fig. 2 (c). With prolonged soaking time, no Ga ions were detected in the 0 wt% group. For the other scaffold groups, the release of Ga ions generally decreased over time, with the 15 wt% Ga-MBG/TCP composite scaffold showing the fastest decrease during the first seven days. The accumulated amounts of Ca and Ga ions are presented in Fig. 2(d) and (e), respectively. With the extension of the immersion time, the cumulative amount of Ca ions released by the four groups of scaffolds gradually increased. Notably, the 10 wt% Ga-MBG/TCP composite scaffold released the most Ca ions, and the 15 wt% Ga-MBG/TCP scaffold released the least. In the cumulative release curve of Ga ions, the 15 wt% Ga-MBG/TCP released the highest cumulative amount of Ga ions, followed by the 10 wt% group. In addition, the Si-ion release of 10 wt% Ga-MBG/TCP and 15 wt% Ga-MBG/TCP initially decreased and then stabilized, whereas the release from the 5 wt% Ga-MBG/TCP remained stable throughout the experiment (Fig. s2).

### 3.4 Biocompatibility of Ga-MBG/TCP composite scaffold

To evaluate the biocompatibility of biomaterials with organisms, we investigated the effect of the Ga-MBG/TCP composite scaffold on the viability and proliferation of rat bone marrow stromal cells (rBMSCs). In the CCK-8 experiment, significant differences in cell viability were observed in the 15 wt% group on day 3 (Fig. 3b). Live and dead staining assays further revealed a higher proportion of dead cells in the 15 wt% group on days 3 and 5 (Fig. 3a–c). Hemolysis tests are key to evaluating the safety of long-term implanted materials [40]. Therefore, we performed a hemolysis test on these stents. The results indicated that red blood cells from Sprague-Dawley (SD) rats treated with water showed extensive lysis, whereas those treated with phosphate-buffered saline (PBS) retained their bright red color (Fig. 3d and e). The scaffold with a concentration of 15 wt% in the experimental group exhibited a higher dissolution rate than the other groups. The results showed that at higher concentrations, the Ga-MBG/TCP composite scaffold had a certain inhibitory effect on biological activity. However, none of the components exhibited obvious biotoxicity.

**Fig. 3:**
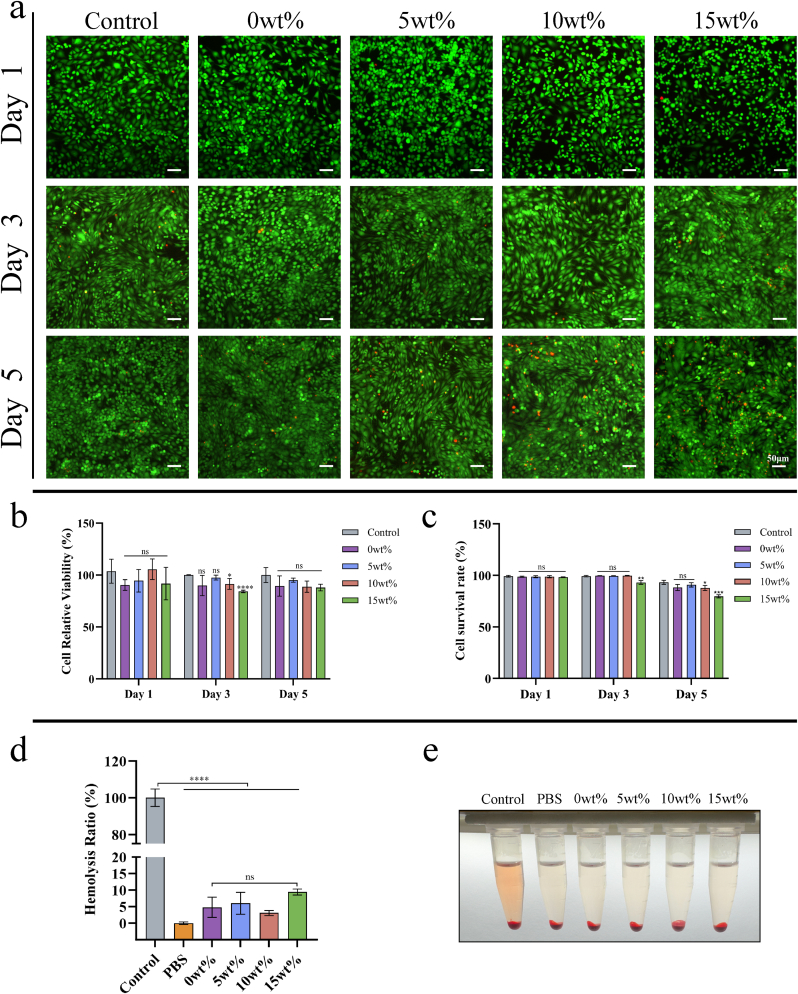
Cell compatibility experiment. (a) Live and dead staining of bone marrow mesenchymal stem cells after co-culture with scaffolds for 1, 3, and 5 days. (b, c) Assessment of cell viability. (d, e) Hemolysis test. Data are show as mean ± SD (n = 3). ∗p < 0.05; ∗ ∗p < 0.01; ∗ ∗ ∗p < 0.001, and ∗ ∗ ∗ ∗p < 0.0001.

The compatibility of gallium-based biomaterials has been extensively studied. However, the clinical translation of metallic gallium and its composites remains challenging due to its high concentration and potential cytotoxicity for wide applications [41]. The TCP component within the composite scaffold successfully underwent clinical translation due to its favorable biological activity. Similarly, MBG has been extensively utilized in tissue engineering research owing to its advantageous biological properties [[42], [43], [44]]. Regulating the concentration of the released Ga ions from these biomaterials is critical to optimizing their promotion of bone regeneration [45], suppression of osteoclast activity, and maintenance of biocompatibility. In this study, the Ga-MBG scaffold at a concentration of 10 wt% demonstrated excellent biocompatibility within its effective concentration range. This finding aligns with the periodic ion release results, where gallium ions were stably released alongside a significant amount of calcium ions. This balance supports both biocompatibility and bone regeneration.

### 3.5 Promotion of osteoblast formation by Ga-MBG/TCP composite scaffolds

To investigate the effect of different concentrations of Ga-MBG/TCP composite scaffolds on bone regeneration *in vitro*, we performed co-culture experiments using scaffolds with varying concentrations. ALP staining after 7 days of osteogenesis induction and alizarin red staining at 14 days revealed that the staining intensities in the 5 wt% and 10 wt% groups were greater than those in the other groups, with the 10 wt% group demonstrating the highest staining intensity (Fig. 4a–c). These results were confirmed by quantitative analysis (Fig. 4b–d). In addition, after 7 days of co-culture, we tested representative markers closely related to osteoblast differentiation; the immunofluorescence assay for BMP-2 (Fig. 4e and f) and fluorescence quantitative PCR assays for BMP-2, RUNX2, and OCN (Fig. 4g, h, i) showed that expression levels in the 10 wt% group were significantly upregulated in each group compared to the other groups.

**Fig. 4:**
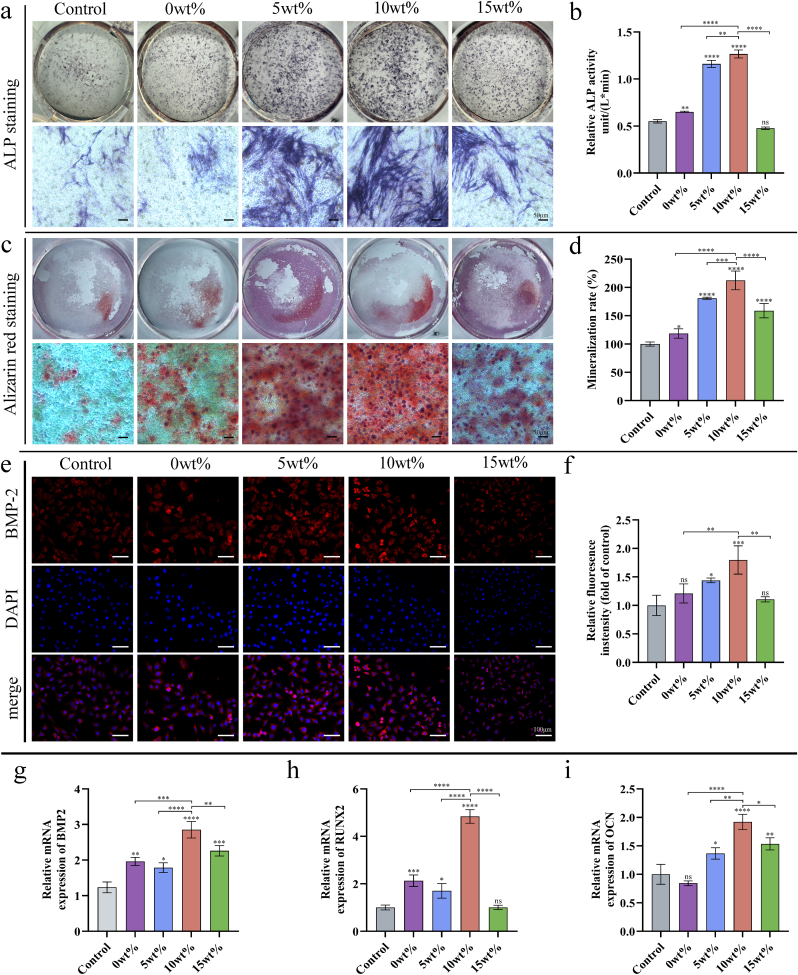
Osteogenic differentiation analysis of bone marrow mesenchymal stem cells after co-culture of scaffolds with different concentrations in osteogenic induction medium. (a, b) ALP staining and ALP activity at 7d. (c, d) Quantification of alizarin red staining and calcium formation at 14 d. (e) Fluorescence staining images of *in vitro* expression of RUNX2. (f) Immunofluorescence semi-quantitative analysis of BMP2. (g–i) Relative mRNA expression levels of osteogenic differentiation-related markers (BMP2, RUNX2 and OCN) in rBMSCs at 7 d. These data are relative to the average level of GAPDH and normalized to the expression level in the Control group. Data are show as mean ± SD (n = 3). ∗p < 0.05; ∗ ∗p < 0.01; ∗ ∗ ∗p < 0.001, and ∗ ∗ ∗ ∗p < 0.0001.

Our results show that β-TCP alone can promote bone regeneration to a certain extent compared to the control group. However, this effect is further strengthened when combined with Ga-MBG within an optimal concentration range. Previous studies have reported that excessively high concentrations of Ga inhibit the osteogenic activity of mesenchymal stem cells. According to our periodic ion release results, high concentrations of Ga reduced Ca ion release, which negatively impacted bone regeneration. This suggests the feasibility of designing a platform that leverages Ga-ion release to promote bone regeneration. According to Kurtuldu et al., suitable concentrations of Ga ions promote cell viability on MBG platforms [24]. However, β-TCP provides a supplementary source of calcium ions needed for bone regeneration. Together, these components synergistically modulated the microenvironment of bone regeneration, effectively strengthening osteogenesis [46,47].

### 3.6 Migration effect of Ga-MBG/TCP composite scaffold on osteoblast formation

**Fig. 5:**
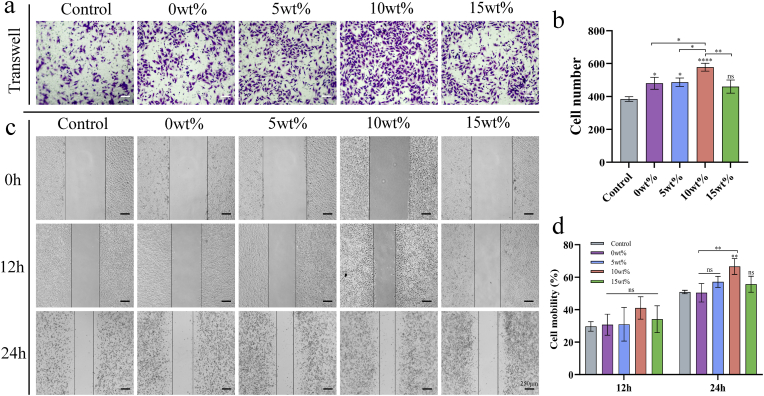
Analysis of the migration of bone marrow mesenchymal stem cells by different concentrations of scaffold extracts. (a) Transwell analysis at 24 h. (b) Semi-quantitative analysis of transwell. (c) Cell scratch experiments at 12 h and 24 h. (d) Semi-quantitative analysis of scratch experiments. Data are show as mean ± SD (n = 3). ∗p < 0.05; ∗ ∗p < 0.01; ∗ ∗ ∗p < 0.001, and ∗ ∗ ∗ ∗p < 0.0001.

Osteoblast migration is a critical physiological process essential for bone tissue regeneration. Sustaining and enhancing the migratory capacity of osteoblasts is critical for treating conditions such as osteoporosis. To investigate the migratory effects of Ga-MBG/TCP composite scaffolds, crystal violet staining and scratch assays were performed. The findings indicated a significant upregulation of cell migration at a concentration of 10 wt% compared to the other groups (Fig. 5).

Our results showed that the 10 wt% Ga-MBG/TCP scaffold most effectively promoted cell migration. Gallium ions incorporated into scaffold materials such as β-TCP attract surrounding mesenchymal stem cells to migrate toward bone defect sites. According to Yu et al., the release of Ga ions at this concentration promotes the migration of osteoblasts through biological effects involving the TRPM7/Akt signaling pathway [48]. The addition of MBG facilitates the sustained release of Ga ions, ensuring prolonged stimulation of cell migration [49].

### 3.7 Biological signal analysis of osteoblast formation using the Ga-MBG/TCP composite scaffolds

**Fig. 6:**
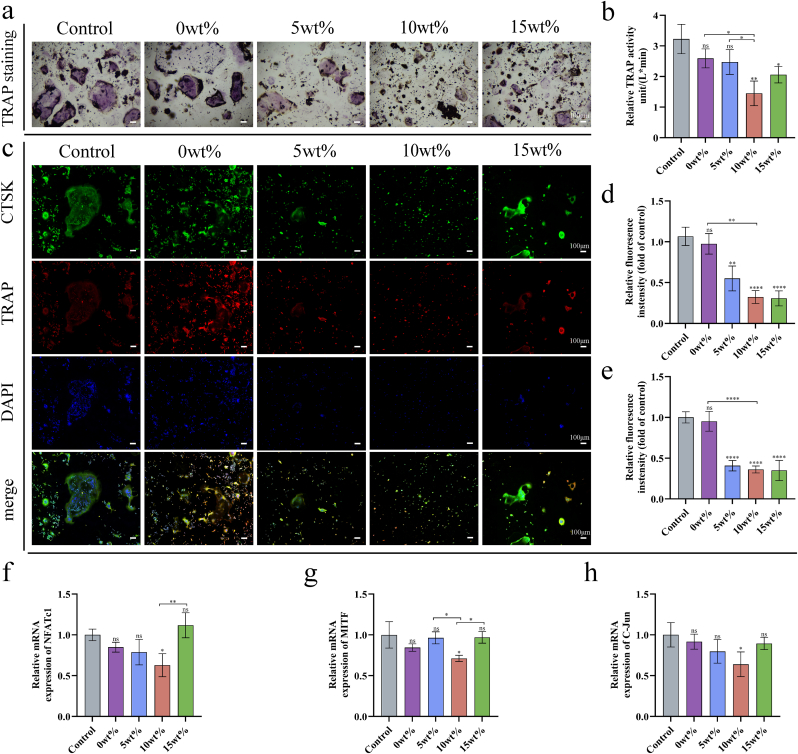
Transcriptome analysis of bone marrow mesenchymal stem cells with scaffolding action. (a) PCA of all samples. (b) Thermographic analysis. (c) Plot of volcanoes compared to control groups. (d) GO enrichment analysis of the scaffold group versus the control group. (d, f) KEGG enrichment analysis in DEG scaffold group compared with control group. (g–i) PPI network analysis.

To evaluate the impact of Ga-MBG/TCP scaffolds on BMSCs at the transcriptomic level, RNA-seq analysis was performed on the control and 10 wt% Ga-MBG/TCP groups after one week of co-culture. The gene expression profiles of all samples were subjected to principal component analysis (PCA) for a deeper transcriptome analysis (Fig. 6a). The sample group correlation coefficients were satisfactory (R^2^ > 0.90, n = 3) (Fig. s3), as shown in the DEG heatmap (Fig. 6b). Compared with the control group, 601 DEGs were downregulated and 981 were upregulated in the 10 wt% group (Fig. 6c). Gene Ontology (GO) and Kyoto Encyclopedia of Genes and Genomes (KEGG) enrichment analyses were used to explore the functions of these DEGs in biological processes, cellular components, molecular functions, and related pathways. GO analysis revealed that the 10 wt% group was enriched in monocarboxylic acid biosynthesis, actin cytoskeleton, and transcription activator activity compared with the control (Fig. 6d). KEGG analysis showed upregulated chemokine and FoxO signaling pathways and downregulated PPAR signaling pathways in the 10 % group (Fig. 6e and f). PPI network analysis showed that the 10 wt% group mainly involved chemokines, such as Cxcl12 and Cxcl10, whereas the downregulated network was associated with serine/threonine protein kinases, particularly PLK1 and PLK3.

Transcriptome analysis showed that the chemokine signaling pathway was upregulated, indirectly affecting osteogenesis and osteoclastogenesis. Chemokines bind to osteoblast surface chemokine receptors (mostly G protein-coupled receptors) to activate the phosphatidylinositol 3-kinase (PI3K)–protein kinase B (Akt) pathway and regulate actin rearrangement to promote migration [50,51]. Akt signaling also promotes β-catenin accumulation by inhibiting glycogen synthase kinase-3β (GSK-3β). Along with the activation of the ERK kinase in the MAPK pathway by chemokines, this process enhances osteoblast proliferation [52,53]. The FoxO signaling pathway, upregulated in the 10 wt% group, is known to promote bone regeneration through FoxO-related genes [54], FoxOs also directly affect the formation and function of osteoclasts [55]. In osteoclasts, the human macrophage colony-stimulating factor (M-CSF) and the receptor activator of nuclear factor κB ligand (RANKL) initiate Akt signaling, thereby facilitating cell proliferation and differentiation while concurrently inhibiting apoptosis. This process may involve the suppression of Akt-mediated osteoclast apoptosis through phosphorylation of FoxO proteins [[56], [57], [58]]. In contrast to AKt action, Mst1 kinase facilitates osteoclast apoptosis by inducing transcriptional activation through FoxO phosphorylation [59]. Downregulation of the PPAR signaling pathway can activate genes associated with adipogenesis, while inhibiting genes involved in osteoblast differentiation, such as Runx2. This modulation predisposes bone marrow-derived stem cells to differentiate into adipocytes, thereby diminishing bone formation [60]. Concurrently, downregulation of serine/threonine protein kinase (PIK1 and PIK3)-related networks impedes osteoclast proliferation and differentiation, disrupting activated transcription factors, cytoskeletal rearrangements, and cell cycle progression [61].

### 3.8 Inhibitory effect of Ga-MBG/TCP composite scaffold on RANKL-induced osteoclastogenesis

**Fig. 7:**
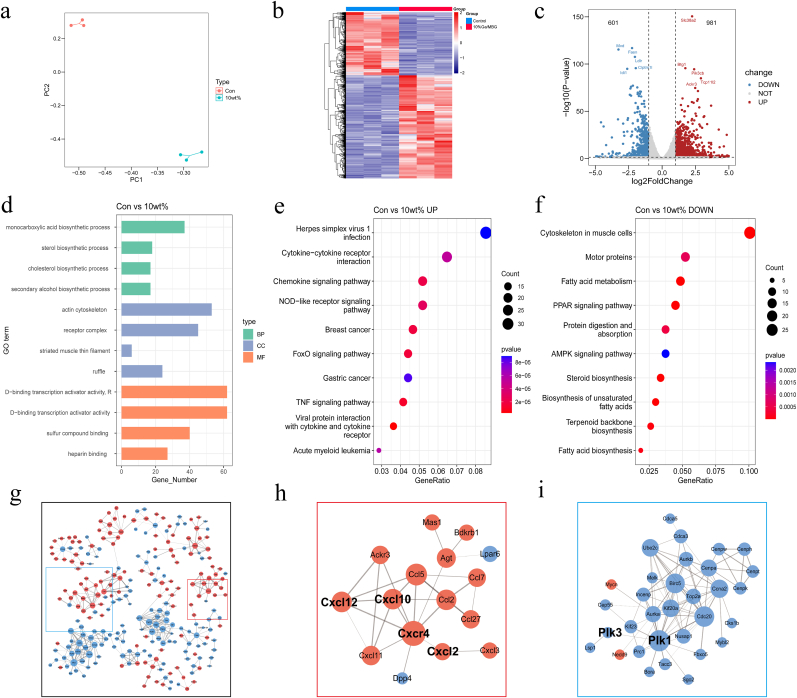
Osteoclasts of RAW differentiated in scaffold extract medium supplemented with indicated concentrations of RANKL. (a) TRAP staining of cells. (b) TRAP activity assay. (c) Fluorescence staining images of *in vitro* expression of CTSK and TRAP. (d) Immunofluorescence semi-quantitative analysis of CTSK. (e) Immunofluorescence semi-quantitative analysis of CTSK. (f–h) Relative mRNA expression levels of osteoclastic differentiation-related markers (NFATc1, MITF, and C-Jun) in rBMSCs at 7 d. These data are relative to the average level of GAPDH and normalized to the expression level in the Control group. Data are show as mean ± SD (n = 3). ∗p < 0.05; ∗ ∗p < 0.01; ∗ ∗ ∗p < 0.001, and ∗ ∗ ∗ ∗p < 0.0001.

To evaluate the effect of the Ga-MBG/TCP composite scaffold on osteoclastogenesis, we tested its cytotoxicity against RAW264.7 cells. Except for the Ga-MBG/TCP composite scaffold at a concentration of 15 wt%, which showed relatively low bioactivity, all other groups demonstrated biocompatibility within the effective range (Fig. s4). Subsequently, each group was evaluated for RAW264.7 with TRAP staining. The control and 0 wt% groups had a higher number of large multinucleated cells characteristic of osteoclast differentiation, while the 10 wt% group showed a significant reduction in TRAP-positive cells (Fig. 7a). Additionally, tartrate-resistant acid phosphatase detection revealed that the acid phosphatase content in each group in the 10 wt% group was downregulated compared with other groups (Fig. 7b). On the 7th day of osteoclast induction, immunofluorescence and fluorescence quantitative PCR were performed to detect representative markers closely related to osteoclast differentiation (Fig. 7c), such as TRAP, cathepsin K, NFATc1, and C-Jun. Quantitative analysis of these markers demonstrated that the 10 wt% Ga-MBG/TCP scaffold significantly suppressed the expression of these markers compared to the control and 0 wt% groups (Fig. 7d–h). These results indicate that the addition of Ga into the composite scaffold effectively inhibits the occurrence of osteoclasts, while β-TCP alone has no obvious effect on the differentiation of osteoclasts. Combined with the results of osteoblast differentiation, it can be concluded that the 10 wt% Ga-MBG/TCP composite scaffold effectively balances biotoxicity, bone regeneration, and osteoclast inhibition. Furthermore, this study confirms that Ga inhibits osteoclast resorption *in vitro* without adversely affecting osteoblast function, reinforcing its potential as an anti-osteoporotic semi-metallic element.

According to Kurtuldu et al., Ga ions effectively prevent bone resorption in clinical studies and are effective in treating osteoporosis and cancer-related hypercalcemia. During bone resorption, osteoclasts degrade bone tissue, releasing minerals such as calcium and phosphorus [24]. Ga appears to reduce bone calcium loss by interfering with osteoclast function, thereby indirectly maintaining bone calcium content. According to previous descriptions, Ga has also been shown to inhibit RANKL-stimulated MAPK activation and suppress the NF-κB pathway in osteoclasts, affecting their differentiation [27,62]. Incorporating β-tricalcium phosphate with Ga in MBG scaffolds provides synergistic effects, making it highly promising for bone regeneration in osteoporosis-related applications.

### 3.9 Ga-MBG/TCP increases bone regeneration and decreases bone resorption in a rat model of osteoporotic skull defects

**Fig. 8:**
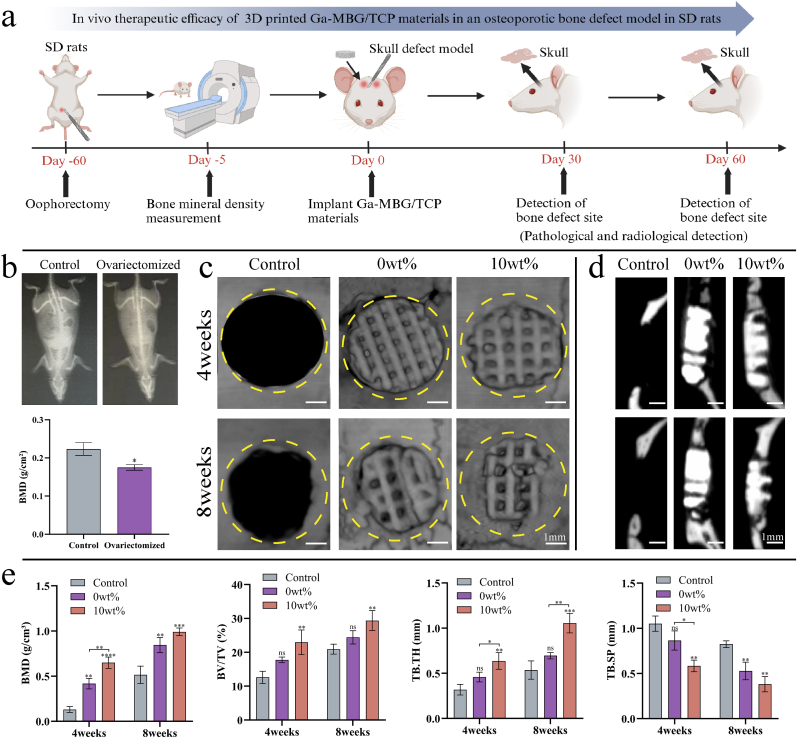
*In vivo* therapeutic effect of Ga-MBG/TCP composite scaffold on osteoporosis rats. (a) Schematic diagram of the *in vivo* experimental procedure in SD rats (Created by BioRender.com). (b) Bone mineral density measurements in SD rats. Microscopic ct images of stent implantation *in vivo*. Cross-sectional (c) and coronal (d) images. (e) Bone mineral density, BV/TV, TB.TH, and TB.SP at 4 and 8 weeks. Data are show as mean ± SD (n = 3). 4 ∗ p < 0.05; ∗ ∗p < 0.01; ∗ ∗ ∗p < 0.001, and ∗ ∗ ∗ ∗p < 0.0001.

**Fig. 9:**
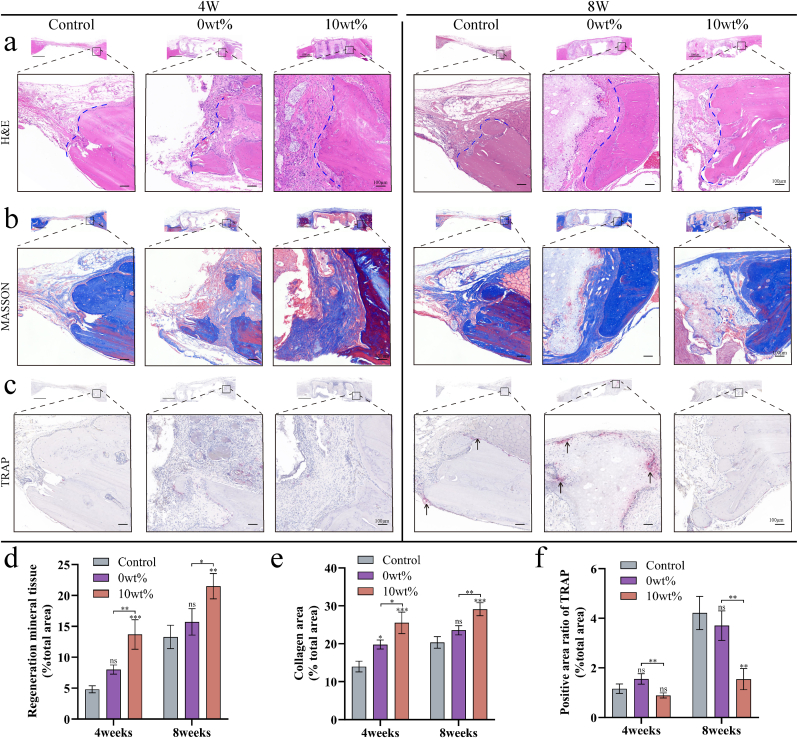
HE, Masson and TRAP staining analysis after implantation of the scaffold into the bone defect site. (a) HE staining at 4 and 8 weeks. (b) Masson staining at 4 and 8 weeks. (b) 4-week and 8-week TRAP staining. The new bone area (d), collagen fiber area (e) and active osteoclasts (f) were analyzed at 4 weeks. Data are show as mean ± SD (n = 3). 4 ∗ p < 0.05; ∗ ∗p < 0.01; ∗ ∗ ∗p < 0.001, and ∗ ∗ ∗ ∗p < 0.0001.

To evaluate the *in vivo* therapeutic effect of Ga-MBG/TCP, we treated SD rats by ovariectomy 60 days before bioscaffold implantation, and bone mineral density measurements were performed 5 days before implantation (Fig. 8a). Compared to the control group, OVX rats had reduced bone mineral density, meeting the osteoporosis criteria in SD rats [53,54] (Fig. 8b). The samples were collected on days 30 and 60 were tested and quantitatively evaluated from the effective area of the removed skull, as shown in Fig. 9c and d. The 10 wt% group demonstrated higher BMD, bone volume to total volume (BV/TV), and trabecular thickness (TB.TH) compared to the other groups (Fig. 8e). Conversely, the 10 wt% group had significantly lower SP values than the other groups, indicating that the Ga-MBG/TCP composite scaffold did not negatively affect bone morphology. Instead, it enhanced osseointegration, osteoblast differentiation, and bone formation.

**Fig. 10:**
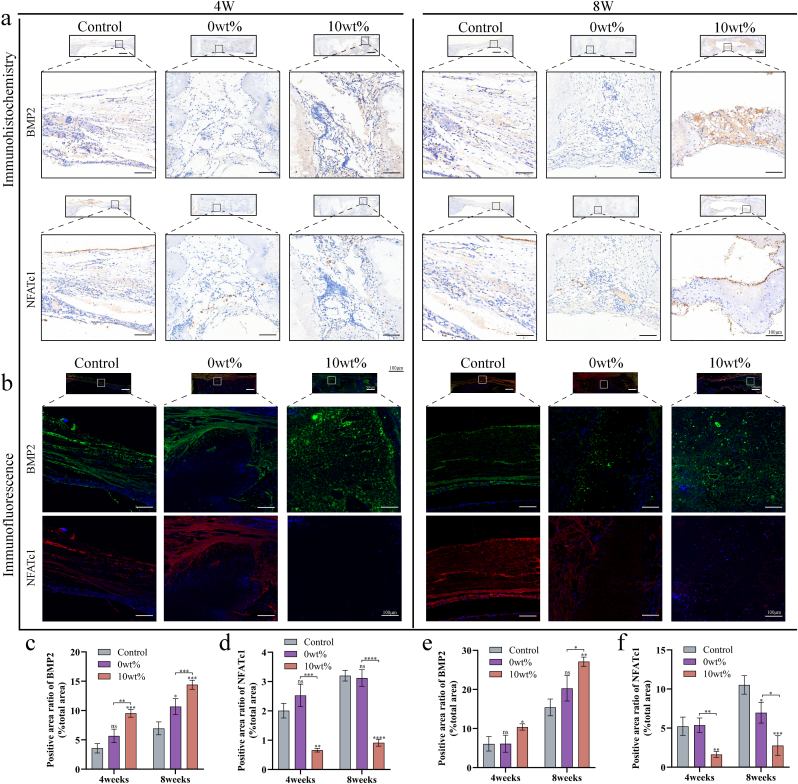
Immunofluorescence and immunohistochemical staining after implantation of bone defect scaffolds. (a) Immunohistochemical images of BMP-2 and NFATc1 at 4 and 8 weeks. (b) Immunofluorescence images of BMP-2 and NFATc1 at 4 and 8 weeks. (c) Semi-quantitative analysis at 4 and 8 weeks of BMP-2 immunohistochemistry. (d) Semi-quantitative analysis at 4 and 8 weeks of NFATc immunohistochemistry. (e) Semi-quantitative analysis of BMP-2 immunofluorescence intensity at 4 and 8 weeks. (f) Semi-quantitative analysis of NFATc1 immunofluorescence intensity at 4 and 8 weeks. Data are show as mean ± SD (n = 3). 4 ∗ p < 0.05; ∗ ∗p < 0.01; ∗ ∗ ∗p < 0.001, and ∗ ∗ ∗ ∗p < 0.0001.

A series of histological analyses were performed along with radiological assessment to verify the results of the microscopic CT reconstruction. Similar to the imaging results, HE and MASSON staining (Fig. 9a and b) showed that the signs of bone regeneration were most obvious in the Ga-MBG/TCP group. TRAP staining showed a higher number of osteoclasts in the blank control group and 0 wt% groups, whereas the Ga-MBG/TCP group exhibited fewer positive osteoclasts (Fig. 9c). These results were further supported by semiquantitative analyses (Fig. 9d, e, f). In addition, as shown in Fig. 10, we performed immunohistochemical and immunofluorescence assays on markers of bone regeneration and osteolysis. In the Ga-MBG/TCP composite scaffold group, the bone regeneration marker BMP-2 was significantly upregulated, while NFATC1, an osteolysis marker, was minimally expressed compared to the other groups (Fig. 10c–f). BMP-2 induces MSCs to differentiate into osteoblasts. BMP-2 binding to MSC receptors triggers intracellular signaling that induces osteoblast-specific markers such as ALP and OCN, initiating osteogenesis [63]. NFATc1 is crucial for transforming osteoclast precursors into mature osteoclasts and enhancing the expression of osteoclast-specific genes such as TRAP and cathepsin K. The products of these genes are essential for bone resorption by osteoclasts [64,65]. Radiological and histopathological studies using animal models have shown that Ga-MBG/TCP composite scaffolds effectively treat osteoporotic bone defects. The 3D porous structure and mechanical properties of the scaffold supported bone growth and physiological loading, as confirmed by micro-computed tomography and histological evaluations. The release of Ga ions was shown to modulate cell behavior and bone metabolism, with the 10 wt% group exhibiting high bone formation and low resorption *in vivo*.

**Fig. 11:**
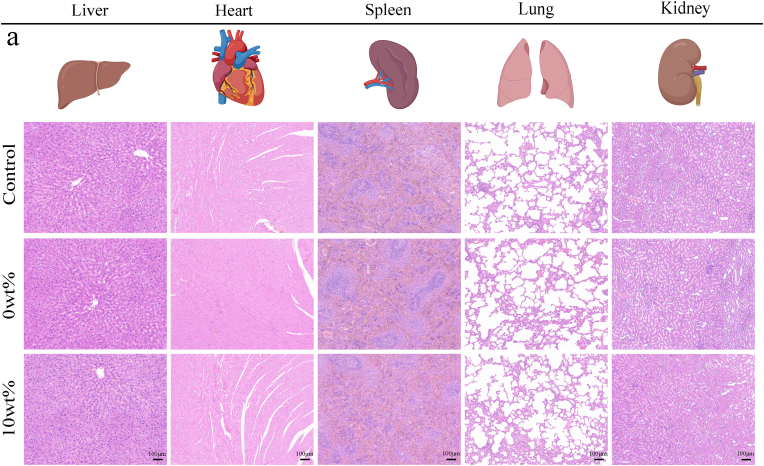
*In vivo* biosafety determination of Ga-MBG/TCP composite scaffolds. (a) Representative H&E staining images of important organs (liver, heart, spleen, lung, kidney) of each group (Created by BioRender.com).

We also evaluated the biosafety of 10 wt% Ga-MBG/TCP composite scaffolds *in vivo*. The tissue structures of the liver, heart, spleen, lungs, and kidneys in each group were roughly normal under H&E staining, and no obvious pathological changes were observed (Fig. 11). A comprehensive assessment of the potential benefits and toxicity risks of gallium-ion-doped biomaterials is essential for their consideration in clinical applications. Consequently, this study substantiated the advantageous properties of the Ga-MBG/TCP composite scaffold in facilitating bone regeneration and mitigating osteolysis. Furthermore, the optimal concentration of the composite was determined, contributing to a deeper understanding of its biodegradability and biocompatibility. These findings suggest that the Ga-MBG/TCP composite scaffold holds significant potential as a candidate for the development of bone repair implants in future clinical applications. However, further systematic investigations are necessary to evaluate the *in vivo* pharmacokinetics and long-term biosafety of these scaffolds. Continued advancements and refinements in related therapeutic strategies are expected to accelerate their application in tissue engineering.

## 4 Conclusions

We used a modified self-conversion method to prepare Ga-doped glass (Ga-MBG) nanoparticles catalyzed by ammonia water and incorporated them into a TCP matrix to prepare Ga-MBG/TCP scaffold materials with different contents by 3D printing. The resulting scaffold exhibited a 3D interconnected porous structure and excellent mechanical properties. Kinetic experiments demonstrated its effective control over gallium ion release. Three groups with different concentrations of Ga-MBG were established, and biocompatibility experiments determined concentrations within an effective range. *In vitro* experiments confirmed the effectiveness of the Ga-MBG/TCP composite scaffold, highlighting its significant osteogenic ability and inhibitory effects on osteoclastogenesis. In addition, we analyzed the biological signals related to osteogenesis and osteoclastic activity throughout the scaffold. To verify the effect of the Ga-MBG/TCP composite scaffold *in vivo*, we employed an osteoporotic rat skull defect model to simulate an osteoporotic bone defect microenvironment. The results showed that the 10 wt% Ga-MBG/TCP composite scaffold demonstrated excellent biocompatibility, promoted the formation of new bone at the implant-bone interface, and reduced osteolysis. We explored the optimal concentration of Ga-MBG implants, which achieved a balance between biological activity, promoted bone regeneration, and reduced osteolysis. This composite scaffold not only provides a reference for the optimal concentration of gallium ions for bone regeneration research but also offers applications in the treatment of osteoporotic bone defects.

## CRediT authorship contribution statement

**Hanrui Xi:** Writing – review & editing, Writing – original draft, Methodology, Investigation. **Xihao Jiang:** Writing – review & editing, Writing – original draft, Methodology, Investigation. **Shilang Xiong:** Software, Methodology. **Yinuo Zhang:** Validation, Software. **Jingyu Zhou:** Resources, Project administration, Funding acquisition, Data curation. **Min Liu:** Visualization, Validation. **Zhigang Zhou:** Methodology, Investigation. **Chengyu Zhang:** Methodology, Investigation. **Shiwei Liu:** Resources, Funding acquisition. **Zhisheng Long:** Resources, Funding acquisition. **Jianguo Zhou:** Supervision, Funding acquisition, Conceptualization. **Guowen Qian:** Supervision, Funding acquisition, Conceptualization. **Long Xiong:** Supervision, Funding acquisition, Conceptualization.

## Funding sources

10.13039/501100004479Jiangxi Provincial Natural Science Foundation under Grant (20224ACB206012), Science and Technology Project of 10.13039/501100020205Jiangxi Provincial Health Commission (202120005), Municipal scientific research project of Ganzhou Municipal Health Commission (202013), Ganzhou science and technology plan project (2022--YB1395), 10.13039/501100001809National Science Foundation of China under Grant (32360232), 10.13039/501100004479Jiangxi Provincial Natural Science Foundation under Grant (20232BAB216052), 10.13039/501100004479Jiangxi Provincial Natural Science Foundation under Grant (20242BAB25453), 10.13039/501100001809National Science Foundation of China under Grant (32360232), 10.13039/501100004479Jiangxi Provincial Natural Science Foundation under Grant (20232BAB216052 and 20242BAB23059).

## Declaration of competing interest

The authors declare that they have no known competing financial interests or personal relationships that could have appeared to influence the work reported in this paper.

## Figures and Tables

**figs1:**
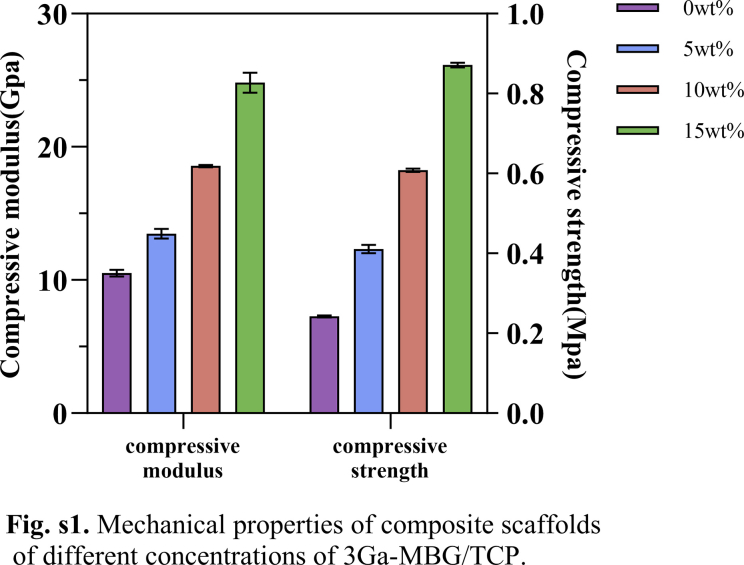


**figs2:**
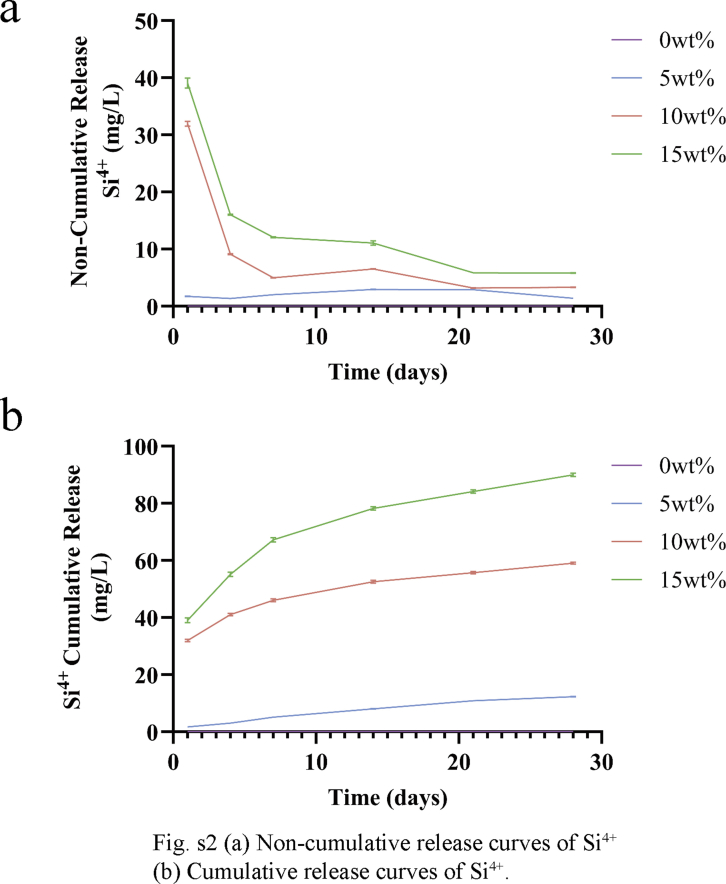


**figs3:**
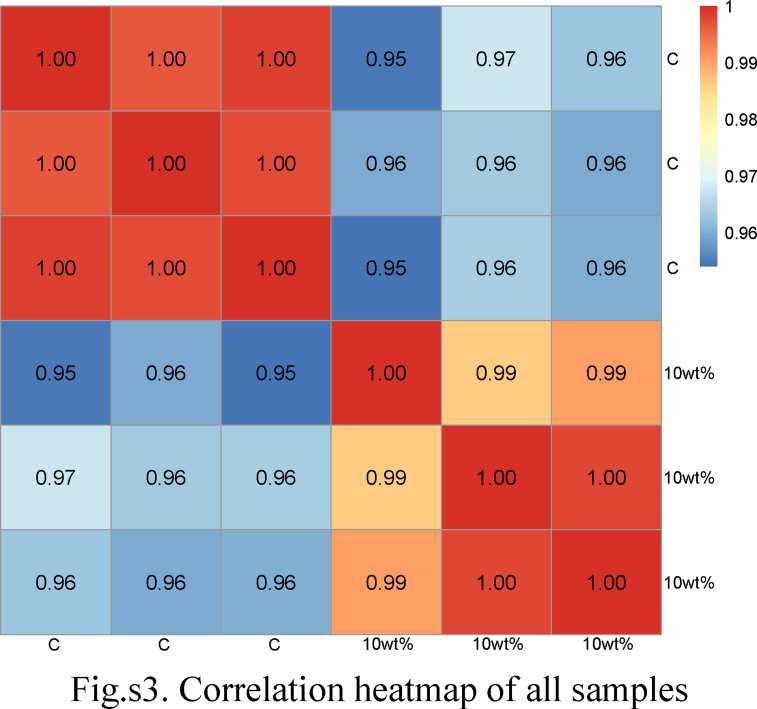


**figs4:**
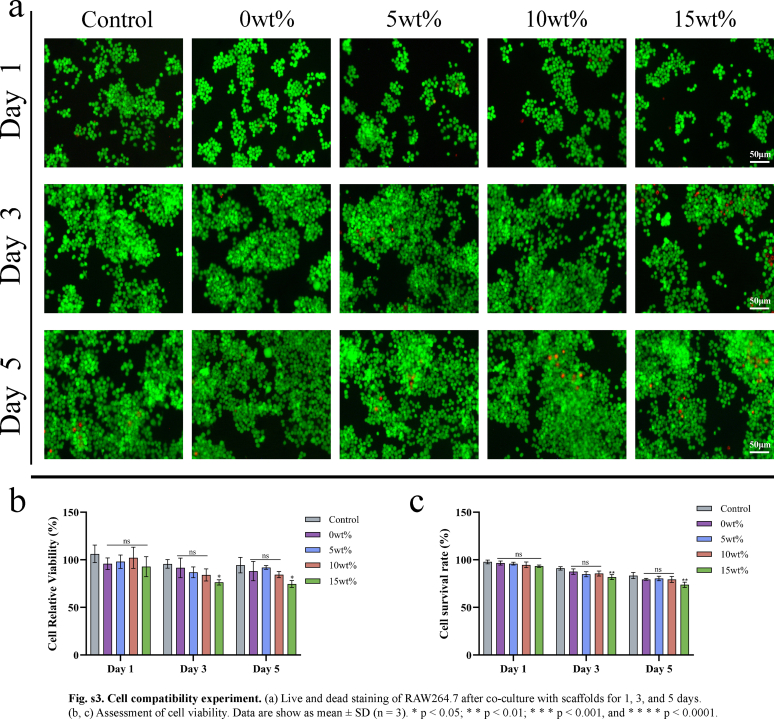


## Data Availability

Data will be made available on request.

## References

[1] Gass M., Dawson-Hughes B. (2006). Preventing osteoporosis-related fractures: an overview. Am. J. Med..

[2] Morin S.N. (2013). Temporal trends in the incidence of osteoporotic fractures. Curr. Osteoporos. Rep..

[3] Johnell O., Kanis J.A. (2006). An estimate of the worldwide prevalence and disability associated with osteoporotic fractures. Osteoporos. Int..

[4] Hernlund E. (2013). Osteoporosis in the European Union: medical management, epidemiology and economic burden. A report prepared in collaboration with the International Osteoporosis Foundation (IOF) and the European Federation of Pharmaceutical Industry Associations (EFPIA). Arch. Osteoporosis.

[5] LeBoff M.S. (2022). The clinician's guide to prevention and treatment of osteoporosis. Osteoporos. Int..

[6] Kammerlander C. (2013). Principles of osteoporotic fracture treatment. Best Pract. Res. Clin. Rheumatol..

[7] Lei C. (2023). Advances in materials-based therapeutic strategies against osteoporosis. Biomaterials.

[8] Zhou J. (2023). Customized additive manufacturing in bone scaffolds-the gateway to precise bone defect treatment. Research (Wash D C).

[9] Li C. (2023). Continuously released Zn(2+) in 3D-printed PLGA/β-TCP/Zn scaffolds for bone defect repair by improving osteoinductive and anti-inflammatory properties. Bioact. Mater..

[10] Zhao M. (2022). A bioactive poly(ether-ether-ketone) nanocomposite scaffold regulates osteoblast/osteoclast activity for the regeneration of osteoporotic bone. J. Mater. Chem. B.

[11] Salazar V.S., Gamer L.W., Rosen V. (2016). BMP signalling in skeletal development, disease and repair. Nat. Rev. Endocrinol..

[12] Tan L. (2013). Biodegradable materials for bone repairs: a review. J. Mater. Sci. Technol..

[13] Wu Q. (2022). Strontium-incorporated bioceramic scaffolds for enhanced osteoporosis bone regeneration. Bone Res..

[14] Hoppe A. (2013). *In vitro* reactivity of Cu doped 45S5 Bioglass® derived scaffolds for bone tissue engineering. J. Mater. Chem. B.

[15] Zhang Y. (2023). 3D-printed flat-bone-mimetic bioceramic scaffolds for cranial restoration. Research (Wash D C).

[16] Wei L. (2014). A comparative study of Sr-incorporated mesoporous bioactive glass scaffolds for regeneration of osteopenic bone defects. Osteoporos. Int..

[17] Wu C. (2016). Europium-containing mesoporous bioactive glass scaffolds for stimulating in vitro and in vivo osteogenesis. ACS Appl. Mater. Interfaces.

[18] He F. (2016). Comparative study on *in vivo* response of porous calcium carbonate composite ceramic and biphasic calcium phosphate ceramic. Mater. Sci. Eng. C Mater. Biol. Appl..

[19] Liu S. (2023). Engineering of a NIR-activable hydrogel-coated mesoporous bioactive glass scaffold with dual-mode parathyroid hormone derivative release property for angiogenesis and bone regeneration. Bioact. Mater..

[20] Romero-Sánchez L.B. (2018). Copper-containing mesoporous bioactive glass promotes angiogenesis in an *in vivo* zebrafish model. Acta Biomater..

[21] Gupta S., Majumdar S., Krishnamurthy S. (2021). Bioactive glass: a multifunctional delivery system. J. Contr. Release.

[22] Sui B. (2023). Mussel-inspired polydopamine composite mesoporous bioactive glass nanoparticles: an exploration of potential metal-ion loading platform and in vitro bioactivity. ACS Appl. Mater. Interfaces.

[23] Li J. (2021). Ion release behavior of vanadium-doped mesoporous bioactive glass particles and the effect of the released ions on osteogenic differentiation of BMSCs via the FAK/MAPK signaling pathway. J. Mater. Chem. B.

[24] Kurtuldu F. (2022). Gallium containing bioactive materials: a review of anticancer, antibacterial, and osteogenic properties. Bioact. Mater..

[25] Mosina M. (2022). Gallium containing calcium phosphates: potential antibacterial agents or fictitious truth. Acta Biomater..

[26] Rachner T.D., Khosla S., Hofbauer L.C. (2011). Osteoporosis: now and the future. Lancet.

[27] Yang Y. (2023). Graphene oxide/gallium nanoderivative as a multifunctional modulator of osteoblastogenesis and osteoclastogenesis for the synergistic therapy of implant-related bone infection. Bioact. Mater..

[28] Dermience M. (2015). Effects of thirty elements on bone metabolism. J. Trace Elem. Med. Biol..

[29] Gómez-Cerezo N. (2018). The response of pre-osteoblasts and osteoclasts to gallium containing mesoporous bioactive glasses. Acta Biomater..

[30] He F. (2021). Conjunction of gallium doping and calcium silicate mediates osteoblastic and osteoclastic performances of tricalcium phosphate bioceramics. Biomed. Mater..

[31] Qiu C. (2020). Influences of gallium substitution on the phase stability, mechanical strength and cellular response of β-tricalcium phosphate bioceramics. Ceram. Int..

[32] Peter Richardo L. (2022). Preparation and characterization of spray pyrolysized strontium-silver-doped mesoporous bioactive glass micron spheres. J. Aust. Ceram. Soc..

[33] Ciraldo F.E. (2018). Synthesis and characterization of silver-doped mesoporous bioactive glass and its applications in conjunction with electrospinning. Materials.

[34] Li X. (2008). Synthesis and characterization of mesoporous CaO–MO–SiO2–P2O5 (M = Mg, Zn, Cu) bioactive glasses/composites. J. Mater. Chem..

[35] Aina V. (2011). Ga-modified (Si–Ca–P) sol–gel glasses: possible relationships between surface chemical properties and bioactivity. J. Phys. Chem. C.

[36] Luo Y., Zhang T., Lin X. (2022). 3D printed hydrogel scaffolds with macro pores and interconnected microchannel networks for tissue engineering vascularization. Chem. Eng. J..

[37] Lu H. (2021). Current application of beta-tricalcium phosphate in bone repair and its mechanism to regulate osteogenesis. Front. Mater..

[38] Baino F., Caddeo S., Vitale-Brovarone C. (2020). Sintering effects of bioactive glass incorporation in tricalcium phosphate scaffolds. Mater. Lett..

[39] Roohani-Esfahani S.-I., Newman P., Zreiqat H. (2016). Design and fabrication of 3D printed scaffolds with a mechanical strength comparable to cortical bone to repair large bone defects. Sci. Rep..

[40] Braune S. (2019). In vitro thrombogenicity testing of biomaterials. Adv. Healthcare Mater..

[41] Chen S. (2023). Toxicity and biocompatibility of liquid metals. Adv. Healthcare Mater..

[42] Baino F., Fiorilli S., Vitale-Brovarone C. (2016). Bioactive glass-based materials with hierarchical porosity for medical applications: review of recent advances. Acta Biomater..

[43] Ryu H.S. (2004). Magnesia-doped HA/beta-TCP ceramics and evaluation of their biocompatibility. Biomaterials.

[44] Pan C. (2020). The effects of β-TCP on mechanical properties, corrosion behavior and biocompatibility of β-TCP/Zn-Mg composites. Mater. Sci. Eng. C Mater. Biol. Appl..

[45] He F. (2019). Modification of honeycomb bioceramic scaffolds for bone regeneration under the condition of excessive bone resorption. J. Biomed. Mater. Res..

[46] Sadowska J.M. (2019). The effect of biomimetic calcium deficient hydroxyapatite and sintered β-tricalcium phosphate on osteoimmune reaction and osteogenesis. Acta Biomater..

[47] Mofakhami S., Salahinejad E. (2021). Biphasic calcium phosphate microspheres in biomedical applications. J. Contr. Release.

[48] Yu M. (2020). Gallium ions promote osteoinduction of human and mouse osteoblasts via the TRPM7/Akt signaling pathway. Mol. Med. Rep..

[49] Lin D. (2019). Rapid initiation of guided bone regeneration driven by spatiotemporal delivery of IL-8 and BMP-2 from hierarchical MBG-based scaffold. Biomaterials.

[50] Galliera E. (2008). Chemokines and bone remodeling. Int. J. Immunopathol. Pharmacol..

[51] Zhu S. (2024). Cell signaling and transcriptional regulation of osteoblast lineage commitment, differentiation, bone formation, and homeostasis. Cell Discov..

[52] Brylka L.J., Schinke T. (2019). Chemokines in physiological and pathological bone remodeling. Front. Immunol..

[53] Wan L. (2024). PI3K/Akt pathway-mediated enhancement of bone and vascular regeneration by gelatin/hyaluronic acid/exosome composite scaffold in bone tissue engineering. Biomater. Adv..

[54] Ma X. (2020). The roles of FoxO transcription factors in regulation of bone cells function. Int. J. Mol. Sci..

[55] Bartell S.M. (2014). FoxO proteins restrain osteoclastogenesis and bone resorption by attenuating H2O2 accumulation. Nat. Commun..

[56] Wong B.R. (1999). TRANCE, a TNF family member, activates Akt/PKB through a signaling complex involving TRAF6 and c-Src. Mol. Cell.

[57] Kawamura N. (2007). Akt1 in osteoblasts and osteoclasts controls bone remodeling. PLoS One.

[58] Almeida M., Porter R.M. (2019). Sirtuins and FoxOs in osteoporosis and osteoarthritis. Bone.

[59] Reszka A.A. (1999). Bisphosphonates act directly on the osteoclast to induce caspase cleavage of mst1 kinase during apoptosis. A link between inhibition of the mevalonate pathway and regulation of an apoptosis-promoting kinase. J. Biol. Chem..

[60] Li B. (2022). Puerarin improves OVX-induced osteoporosis by regulating phospholipid metabolism and biosynthesis of unsaturated fatty acids based on serum metabolomics. Phytomedicine.

[61] Jang J.S. (2024). PINK1 restrains periodontitis-induced bone loss by preventing osteoclast mitophagy impairment. Redox Biol..

[62] Warrell R.P. (1984). Gallium nitrate inhibits calcium resorption from bone and is effective treatment for cancer-related hypercalcemia. J. Clin. Invest..

[63] Lowery J.W., Rosen V. (2018). The BMP pathway and its inhibitors in the skeleton. Physiol. Rev..

[64] Teitelbaum S.L., Ross F.P. (2003). Genetic regulation of osteoclast development and function. Nat. Rev. Genet..

[65] Boyle W.J., Simonet W.S., Lacey D.L. (2003). Osteoclast differentiation and activation. Nature.

